# First Survey of the Wheat Chromosome 5A Composition through a Next Generation Sequencing Approach

**DOI:** 10.1371/journal.pone.0026421

**Published:** 2011-10-18

**Authors:** Nicola Vitulo, Alessandro Albiero, Claudio Forcato, Davide Campagna, Francesca Dal Pero, Paolo Bagnaresi, Moreno Colaiacovo, Primetta Faccioli, Antonella Lamontanara, Hana Šimková, Marie Kubaláková, Gaetano Perrotta, Paolo Facella, Loredana Lopez, Marco Pietrella, Giulio Gianese, Jaroslav Doležel, Giovanni Giuliano, Luigi Cattivelli, Giorgio Valle, A. Michele Stanca

**Affiliations:** 1 CRIBI Biotechnology Center, University of Padova, Padova, Italy; 2 Bmr-genomics srl, Padova, Italy; 3 CRA Genomics Research Centre, Fiorenzuola d'Arda, Italy; 4 Centre of the Region Haná for Biotechnological and Agricultural Research, Institute of Experimental Botany, Olomouc, Czech Republic; 5 ENEA, TRISAIA Research Center, Rotondella, Italy; 6 ENEA, CASACCIA Research Center, Rome, Italy; 7 Ylichron Srl, Rome, Italy; University of Melbourne, Australia

## Abstract

Wheat is one of the world's most important crops and is characterized by a large polyploid genome. One way to reduce genome complexity is to isolate single chromosomes using flow cytometry. Low coverage DNA sequencing can provide a snapshot of individual chromosomes, allowing a fast characterization of their main features and comparison with other genomes. We used massively parallel 454 pyrosequencing to obtain a 2x coverage of wheat chromosome 5A. The resulting sequence assembly was used to identify TEs, genes and miRNAs, as well as to infer a virtual gene order based on the synteny with other grass genomes. Repetitive elements account for more than 75% of the genome. Gene content was estimated considering non-redundant reads showing at least one match to ESTs or proteins. The results indicate that the coding fraction represents 1.08% and 1.3% of the short and long arm respectively, projecting the number of genes of the whole chromosome to approximately 5,000. 195 candidate miRNA precursors belonging to 16 miRNA families were identified. The 5A genes were used to search for syntenic relationships between grass genomes. The short arm is closely related to *Brachypodium* chromosome 4, sorghum chromosome 8 and rice chromosome 12; the long arm to regions of *Brachypodium* chromosomes 4 and 1, sorghum chromosomes 1 and 2 and rice chromosomes 9 and 3. From these similarities it was possible to infer the virtual gene order of 392 (5AS) and 1,480 (5AL) genes of chromosome 5A, which was compared to, and found to be largely congruent with the available physical map of this chromosome.

## Introduction

Wheat is one of the world's most important crops, but despite its economic importance progress in wheat genomics has been slow due to its large and complex genome. The gene number of wheat is expected to be about 90,000, i.e. approximately three times that of rice, (http://rapdb.dna.affrc.go.jp), while the size of the wheat genome is very large (16,937 Mb/1C, [1]), more than 40 times that of the rice genome. Several studies, coordinated by the International Wheat Genome Sequencing Consortium (http://www.wheatgenome.org) are in progress with the aim of obtaining and characterizing the wheat genome.

One way to reduce the genome complexity and simplify its analysis is to purify single chromosomes using flow cytometry and to perform the analysis at the sub-genomic level [2], [3]. For example, Paux et al. [4] and Bartoš et al. [5] studied the molecular structure of the wheat chromosome 3B and the rye chromosome arm 1RS, respectively, after sequencing ends of BAC clones from chromosome-specific libraries. When flow-sorted chromosomes are subjected to low-coverage massively parallel DNA sequencing, a snapshot of single chromosomes can be produced even in species with a large genome. In the first study of this type, Mayer et al. [6] characterized repetitive elements and genes in the barley chromosome 1H, identified syntenic regions with genomes already sequenced and produced a linearly ordered high-resolution gene inventory of the chromosome.

The repetitive landscape of large plant genomes has proven to be a major bottleneck for whole genome sequencing. Understanding the role and dynamics of Transposable Elements (TEs) may shed light on key aspects such as genome restructuring and speciation, stress resistance and epigenetics [7], [8], [9]. Gaining an early insight with respect to TE composition may also prove useful to counteract technical issues arising during genome assembly and finishing. Recently, several studies have started to elucidate the structure and complexity of graminae (*Poaceae*) mobile elements, showing that their abundance appears to contribute significantly to genome sizes. According to a recent genome draft [10], nearly 85% of the 2.3-gigabase maize genome is composed of TEs, while its close relative *Sorghum bicolor*, with a smaller genome size (750Mb), has only 61% of TEs. Also, the small genome of the model grass *Brachypodium distachyon* (355Mb), contains only 28% TEs [11]. The closest wheat relative for which estimates on repeat content have been obtained is barley (5.1Gb genome size), with proportions ranging from 70% to 74.5% [6], [12]. Early estimates based on limited datasets suggested about 80% of repeated elements in the wheat genome [13].

The close syntenic relationships among *Poaceae* are well known [14]. However, knowledge on the gene content of single chromosomes will allow detailed inferences on syntenic relations between wheat and other *Triticeae* with sequenced genomes (rice, *Brachypodium*, sorghum). For instance, the integration of low-coverage shotgun sequencing information of barley chromosome 1H with the collinear gene order of orthologous rice and sorghum genes has allowed Mayer et al. [6] to propose a virtual sequence-based gene order map of an entire *Triticeae* chromosome.

MicroRNAs (miRNAs) are short non-protein-coding RNAs produced by Dicer or Dicer-like enzymes which catalyze excision from longer precursors. They are present in both animals and plants, although in these taxa substantial differences are present with respect to their biogenesis and mechanism of action [15]. Besides acting as regulators of development and morphogenesis, plant miRNAs have been recently associated with biotic and abiotic stress responses [15], [16], [17]. Based on sequence similarity, miRNAs have been classified in families whose members can show a high level of conservation across many plant species (conserved miRNAs). Thanks to this fact, bioinformatics can be used in a straightforward way to discover homologs of known miRNAs, while the search for novel and species-specific miRNAs is mainly accomplished by lab-based methods [15]. In the *miRBase* [18] release 16 (September 2010) only 42 wheat microRNAs are included [19], although other candidate miRNAs have been proposed [20], [21]. Little is known about the specific chromosome locations of all these miRNAs and no information is available on the content of miRNAs in wheat chromosome 5A.

In this work, we provide a first sequencing survey of the molecular landscape of wheat chromosome 5A. With a size of 827Mb, the chromosome harbors 4.9% of the wheat genome [1] and carries genes controlling important traits such as vernalization requirement (*TaVRT-1* [22]), cold tolerance and abiotic stress tolerance [23], [24], disease resistance (e.g. Fusarium head blight [25]) and domestication traits (e.g. free threshing Q gene [26]). Massively parallel 454 pyrosequencing was used to obtain a 2x coverage of the chromosome and the resulting sequence assembly allowed us to characterize for the first time genes, miRNAs and overall TE content and to infer a virtual gene order and synteny of chromosome 5A with other grass genomes.

## Results and Discussion

### Chromosome arm sorting and amplification of chromosomal DNA

Flow cytometry was used to isolate the short and long arms of wheat chromosome 5A. The arms were sorted as telocentric chromosomes 5AS and 5AL from a double ditelosomic line (dDt5A). The use of telosomic stocks enables the dissection of the huge wheat genome into small parts which facilitates its analysis and mapping [3]. Flow karyotypes obtained after the analysis of liquid suspensions of DAPI-stained mitotic chromosomes had three composite peaks (I, II and III) representing groups of chromosomes, a small peak of chromosome 3B, and two peaks representing the 5AS and 5AL arms (Figure S1). The arms were sorted into five (5AS) and two (5AL) replicates on microscopic slides and FISH analysis indicated 5–15% contamination by other chromosomes. The samples were contaminated by chromatid fragments and chromosomes without a prevalence of a particular chromosome type. One sample of 5AS with 5.3% contamination and one sample of 5AL with 14.4% contamination were selected for sequencing. Approximately 10 ng DNA were obtained after proteinase treatment and column purification from each chromosome arm fraction. Purified DNA was used as a template for multiple-displacement amplification, which yielded 3–6 µg of DNA.

### Sequence assembly

**Figure 1:**
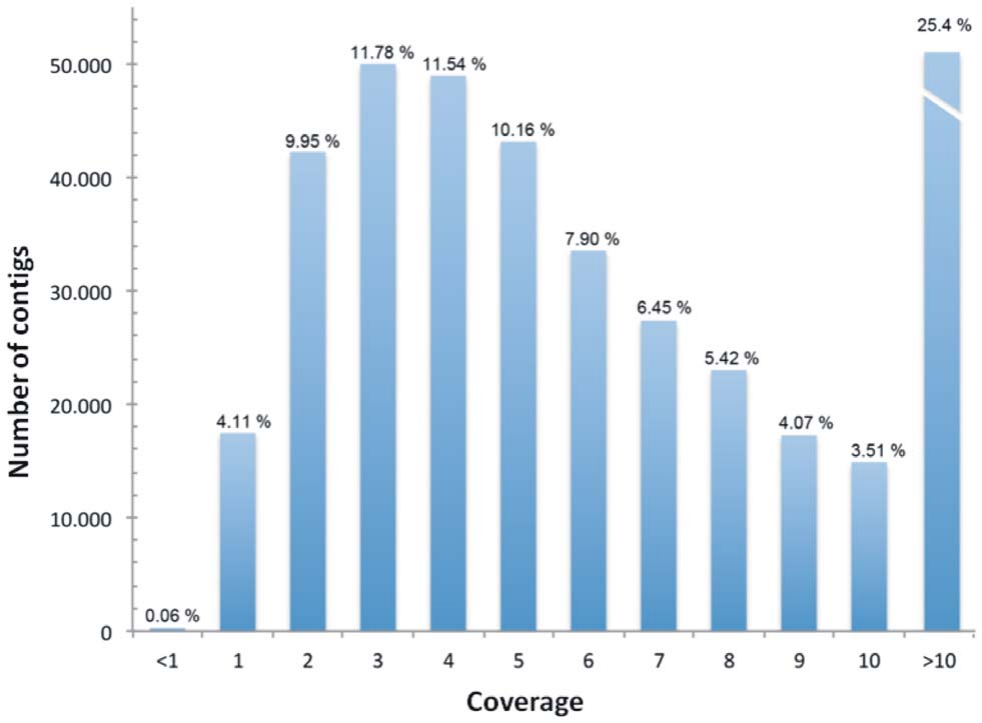
Assembly contig coverage distribution of the whole chromosome. The majority of the contigs show a coverage higher than 10x (the highest covered contig was 733x). These data agree with the high repetitive DNA content. All contigs with coverage higher than 10x are presented in the same class. The real peak of the distribution is between 2x and 3x coverage. This peak confirms the theoretical sequence coverage (theoretical coverage is 2.4x, estimated coverage is 2.3x).

Complete assembly of the chromosome 5A is beyond the scope of the current work, since the low sequence coverage and the high proportion of repetitive elements prevents successful assembly. Nevertheless, a preliminary assembly of sequencing data can provide useful information on chromosome structure. Table 1 reports the main metrics of the 454 sequencing reactions and of the assemblies. Two independent assemblies were performed with the reads obtained from the single arm reactions and then an additional assembly was run on the whole 5A chromosome data set. The results show a low percentage of aligned reads and a large number of short contigs, an expected finding considering the low sequencing coverage. The preliminary chromosome assembly allowed to estimate a coverage of 2.3x and a chromosome size of 857.8Mb, very close to the predicted chromosome size of 827Mb [1]. The total length of the assembled contigs was only 173.2Mb. This small size can be attributed to three main reasons: i) a high number of sequence reads are classified as singletons and therefore do not contribute to the 173.2 Mb; ii) the high number of repeated regions assemble in collapsed contigs [4], as confirmed by the high coverage of a large number of contigs (Figure 1); iii) according to the Poisson distribution, with a 2.3x coverage more than 10% of the genome is expected to be not covered. Finally, the whole 5A genome assembly was used to verify the overlap between the two arms in terms of sequence similarity; 40% of the contigs are composed exclusively of reads from the long arm, 20.7% of reads from the short arm, while 39% of the contigs contain reads from both arms. This overlap is most probably due to repeated regions present on both arms. However, we cannot exclude the possibility that the 5A telosomes were not telocentric but acrocentric, carrying small segments of pericentromeric chromatin from opposite arms.

**Table 1:** Metrics of 454 sequencing reactions and of the three independent assemblies.

Metrics	Short-arm	Long-arm	Whole chromosome
**# 454 Titanium runs**	3	4	7
**# of reads**	2,407,891	3,324,512	5,732,403
**# of bases**	720,256,529	1,257,463,363	1,977,719,892
**AVG sequence length**	299	378	345
**Theoretical coverage**	2.44X	2.35X	2.40X
**# assembled reads**	933,540	1,154,599	1,999,592
**# singleton**	428,594	809,056	1,228,334
**# large contigs (>500 bp)**	37,173	79,117	114,518
**# bases of large contigs**	30,504,362	64,117,733	92,685,645
**AVG large contig size (bp)**	820	810	809
**N50 large contigs (bp)**	803	798	795
**Largest Contig (bp)**	11,639	9,725	9,606
**# all contigs**	159,243	276,221	424,407
**# bases all contigs (bp)**	60,984,419	116,223,592	173,242,208
**Estimated Genome Size (MB)**	182.2	546.4	857.8
**Estimated Coverage**	3.9	2.3	2.3 X

### The repetitive landscape of wheat chromosome 5A

A total of 2,407,891 and 3,324,512 reads obtained from 5AS and 5AL, respectively, were subjected to independent analyses to identify sequences corresponding to repeat elements. The reads were subjected to local BlastN against *Triticeae* genomic repeat sequences (TREP complete database, BlastN; Expect value <10e^−6^). Matching reads were assigned to the “Known Transposable Elements” (Known TEs) group according to the procedure of Wicker et al. [12]. This class accounted for 72.67% (1,749,853 reads) and 71.14% (2,365,036 reads) for 5AS and 5AL, respectively (Table 2). These reads were further assigned to TE families (or subfamilies) according to the nomenclature of Wicker et al. [27]. The number of identified known TE families (or subfamilies) was 276 and 288 for 5AS and 5AL, respectively (see Table S1 for the complete listing of TE families).

**Table 2:** Summary of 454 wheat chromosome 5A long and short arm reads, classified according to major repeat classes.

Groups of repeat elements	Short arm reads	% short arm	Long arm reads	% long arm
**Known TE** (TREP complete database hits)	1,749,853	72.67	2,365,036	71.14
**Novel TE** (PTREP database hits)	59,832	2.48	86,459	2.60
**Repeats (unknown)**	23,419	0.97	282,130	8,49
**Known TE + Novel TE + Repeats**	1,833,104	76.13	2,733,625	82.23
**Genes**	24,721	1.03	41,645	1.25
**Other**	550,066	22.84	549,242	16.52
**Total reads**	2,407,891	100	3,324,512	100

The long and short arm reads were grouped in: known TE families (match at DNA level as indicated by BlastN hits on TREP complete database; Expect value <10e^−6^), Novel TE families (match at protein level indicated by BLASTX hits on protein TREP database; Expect value <10e^−6^), Repeats (uncharacterized repeats belonging to high-coverage contigs not related to genes or other known TE fraction), and Other (leftover reads escaping the previous groups).

Although it has been reported that chromosome DNA amplification does not introduce a significant bias against particular genomic loci [6], [28], some data indicate that chromosome amplification, as conducted in the present study, may result in artificial enrichment in discrete groups of abundant TEs [29], [30]. When comparing data from non-amplified [31] vs. amplified DNA from barley, it was shown that a bias is present with respect to very abundant TE families (Wicker T. and Stein N., personal communication). A survey of the frequency of the main TE families in the non-amplified Chinese Spring whole-genome 5x sequences (http://www.cerealsdb.uk.net/search_reads.htm) indicated a bias for few major TE species present in the 5A dataset. For instance, based on Blast analysis (E-value < = 10e^−5^) *Sabrina* and *Wham* appeared to be two-fold overestimated the 5A dataset, while the opposite was found for *Angela* and *Wis*. No substantial bias could be detected for other, less abundant, species.

Novel TEs, while bearing scarce similarity at the DNA level to known TEs, may nonetheless exhibit substantial similarity to known TEs in the protein-coding regions [31]. Therefore, the reads that did not produce any BlastN match were locally aligned with BlastX (Expect value <10e^−6^) against PTREP. The resulting hits were grouped in the Novel TEs fraction, accounting for 2.48% and 2.60%, short and long arm respectively (Table 2). Again, the non-matching reads were saved for downstream analyses. To estimate additional uncharacterized repeated sequences, a class of “Repeats” was created consisting of reads participating to contigs with high coverage. In fact, given the approximate 2-fold coverage of the snapshot, any coverage higher than 5 is likely to include repeated sequences. This class of repeats was 0.97% and 8.49% for the long and short arm, respectively (Table 2). Overall, wheat 5A repetitive elements, when summing up known TEs, novel TEs and Repeats groups, account for 76.13% (short arm) and 82.23% (long arm) of the snapshot.

### Gene content estimation on wheat chromosome 5A

**Figure 2:**
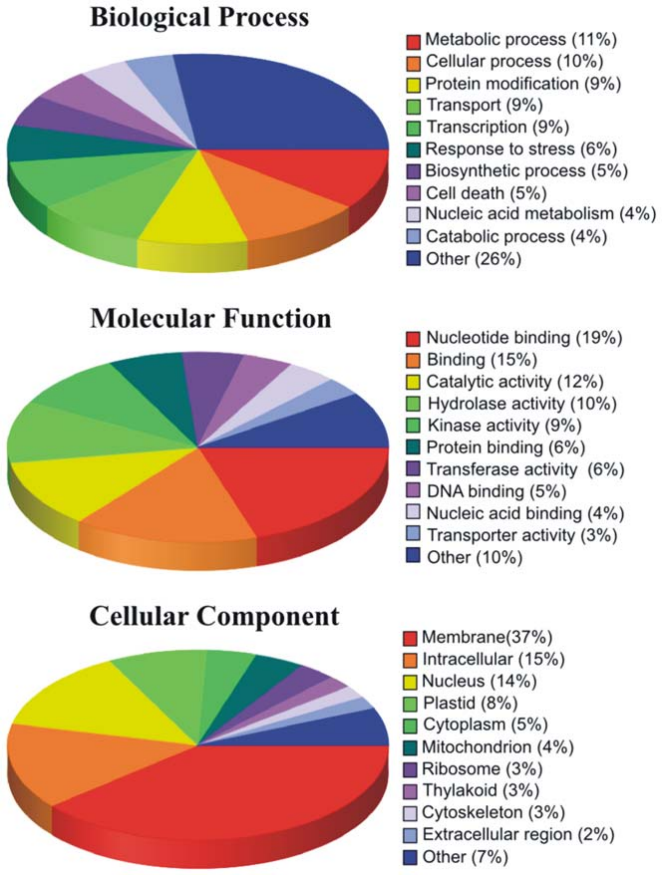
Percentage distribution of the GO entries associated wheat 454 reads for chromosome 5A. The most represented entries within the three ontologies (Molecular function, Biological process and Cellular component) are indicated.

All sequence reads with no similarity to known putative or new repetitive elements were used to characterize the genes and to estimate the gene content of chromosome 5A. The Gene Ontology (GO) annotations obtained by BlastX matches of 454 reads against monocot proteins are shown in Figure 2. The analysis allowed the association of 75,834 reads with 87 terms: 37 Biological Process, 25 Molecular Function and 25 Cellular Component. Biological Process terms were associated to 22,816 reads, Molecular Function categories annotated 44,196 reads, most of which (34%) as “binding” and “DNA binding” function; Cellular Component categories annotated 8,822 terms, 37% of them as “Membrane”.

A similarity search was performed against a subset of UniProt containing only monocotyledon proteins and UniGenes containing all the available Liliopsida ESTs (*Hordeum vulgare, Oryza sativa, Panicum virgatum, Saccharum officinarum, Sorghum bicolor, Triticum aestivum and Zea mays*). The search on the short arm produced 20,108 reads with protein matches and 23,310 reads with EST matches. The same analysis performed on the long arm produced 37,758 reads matching to Uniprot entries and 35,198 reads having similarity to ESTs. The reads matching either to ESTs or proteins were 24,721 for the short arm and 41,645 for the long arm, corresponding to a total length of 7,779,334bp and 16,431,167bp, respectively. Considering the predicted chromosome arm lengths of 295Mb and 532Mb for 5AS and 5AL [1], the coding fraction can be estimated as 1.08% and 1.30% in the short and the long arm, respectively. Considering an average coding sequence length of 2,000 bp [4], the number of genes were estimated at about 1,593 genes on the short arm and 3,495 genes on the long arm, for a total of 5,088 genes for the whole chromosome. This preliminary estimate agrees with the gene content of about 6,000 (1.2%) in the 1Gb chromosome 3B, calculated by BAC end sequencing [4]. However, these estimates should be interpreted with caution as the results depend on partial data sets. For example, Choulet et al. [29] estimated a gene content of about 8,400 in chromosome 3B after sequencing a total of 13Mb BAC contigs.

### Wheat chromosome 5A gene distribution compared to Brachypodium, rice and sorghum genomes

**Figure 3:**
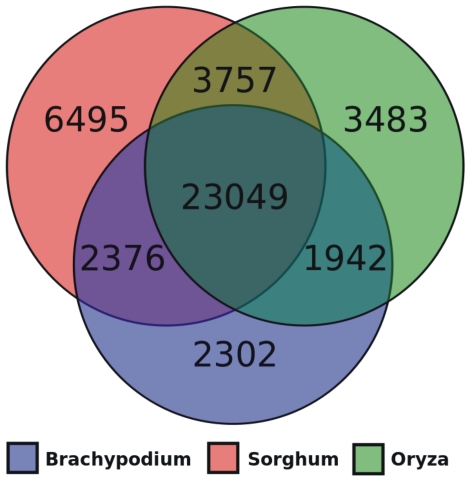
Venn diagram showing wheat read distribution with a significant similarity to *Brachypodium*, rice and sorghum.

**Figure 4:**
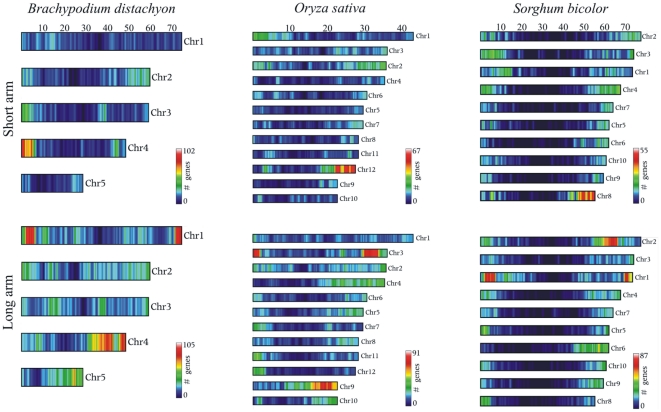
Wheat 5A sequence reads mapped on the genome of *Brachypodium*, rice and sorghum. The genes of *Brachypodium*, rice and sorghum with a significant similarity at the amino acid level with the wheat 5A reads obtained in this work were plotted along their position on the chromosomes of their respective organism. To highlight regions containing a high proportion of putative homologous genes, the average syntenic content was calculated as the number of wheat matching proteins in a window of 1Mb with shift of 20Kb. Note that color scale is specific for each arm and genome, therefore a direct comparison of the gene density between the different genomes is not possible.

**Figure 5:**
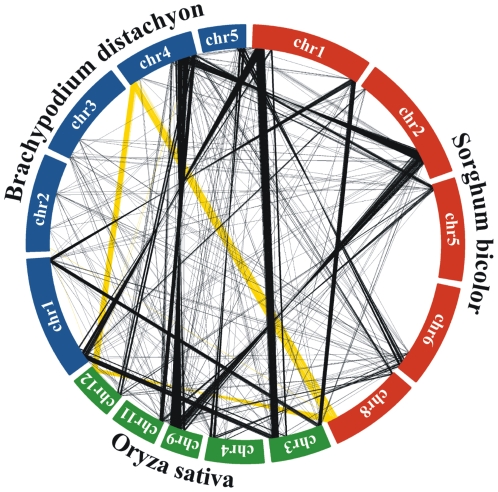
Synteny among *Brachypodium*, rice and sorghum highlighted by wheat 5A read matches. The genes of *Brachypodium*, rice and sorghum showing similarities at the amino acid level with the wheat 5A reads obtained in this work were used to link the chromosomes to highlight the syntenic relationships. Yellow lines refer to the reads from short-arm (5AS), while black lines refer to the reads from long-arm (5AL). Only the genes falling on a region with a gene density higher or equal than 50 genes / Mb were considered (see also Figure 4).

The availability of a 2x sequence coverage of wheat chromosome 5A allows the evaluation of possible syntenic regions shared with the *Brachypodium*, rice and sorghum reference genomes. The analysis was performed at the protein level on the proteomes of the three grass species, using BlastX to search wheat homologous proteins, filtered for transposable elements. The Venn diagram in Figure 3 shows the distribution of the wheat reads with a significant similarity among grass proteins. As expected, the majority of wheat coding sequences found a homologous protein in all three species, reflecting their close phylogenetic relationship [11]. However, 6,495 wheat reads found a homologous protein only in *Brachypodium*, 3,483 in sorghum and 2,302 in rice. The proteins that showed a significant similarity to wheat reads were plotted along their position on the chromosomes of their respective species, highlighting regions containing a high proportion of putative homologous sequences. The average syntenic content was calculated as the number of wheat matching proteins in a window of 1Mb with steps of 20kb. The regions showing synteny to the short and long arms of wheat chromosome 5A are shown in Figure 4 and Figure 5.

Regions of synteny to chromosome 5A, particularly to the long arm, are distributed over many grass chromosomes, suggesting a relatively low conservation of gene order, as reported earlier [32]. The analysis highlights regions of low syntenic gene density towards the end of many chromosomes (green regions in Figure 4). These regions probably result from either genes that really have moved from their original positions or false positive matches due to the alignment between paralogous genes.

Nevertheless, some regions of synteny were identified. The wheat chromosome 5A short arm is closely related to the *Brachypodium* chromosome 4, the sorghum chromosome 8 and the rice chromosome 12. The 5A long arm found syntenic regions on the *Brachypodium* chromosomes 4 and 1, the sorghum chromosomes 1 and 2 and the rice chromosome 9 and 3. These results are in agreement with other comparative studies among grasses, based mainly on RFLP mapping of closely related species [32], [33], [34], [35], [36].

A recent evolution model for cereal genomes proposes a grass ancestor genome with five chromosomes that underwent a whole genome duplication followed by two interchromosomal translocation and fusions that led to an intermediate ancestor with 12 chromosomes [33]. Our results find regions of high synteny between wheat chromosome 5A and rice chromosomes 9 (derived from ancestral chromosome 8) and 12 (derived from ancestral chromosome 11), thus supporting that model.

We used the genomic regions of conserved homologies identified in *Brachypodium*, rice and sorghum, to generate a “genome zipper” [6] for both the short and long arms of the wheat chromosome 5A. The genome zipper method is based on the idea that the virtual gene order in a species can be reconstructed on the basis of the syntenic relationships with closely related species. To build the genome zippers, the *Brachypodium* gene order was used as a reference, considering only the genes of *Brachypodium* having a homologue in at least one of the two other species considered (sorghum and rice). The final 5AS zipper (Table S2) contains 645 genes from the *Brachypodium* chromosome 4 short arm, while the 5AL zipper contains 2,280 genes from both the chromosome 1 and the long arm of chromosome 4.

Each gene of the zipper was associated with an estimate of the reliability of the syntenic region. The resulting vector indicates if the homologous genes from *Brachypodium*, rice and sorghum are located on the expected chromosome and if they can be considered as true orthologous genes as verified by the reciprocal best hit method (see Materials and Methods for details). The 5AS zipper contains 54% of genes located on the syntenic chromosomes, with 81% of them being true orthologs. Similar numbers are found in the 5AL zipper, with 56% of genes located on the right positions and 82% of true orthologs. These values reflect the accuracy of the zipper prediction.

The two reference zippers were used to search for the wheat putative syntenic genes allowing the creation of a virtual gene order for the wheat 5A chromosome. A similarity search of wheat sequences at the nucleotide level identified 392 and 1,480 genes on the 5AS and 5AL zippers, respectively. 55% and the 61% of the 5AS and 5AL wheat sequence matches occur with true orthologous zipper genes located on syntenic chromosomes.

**Figure 6:**
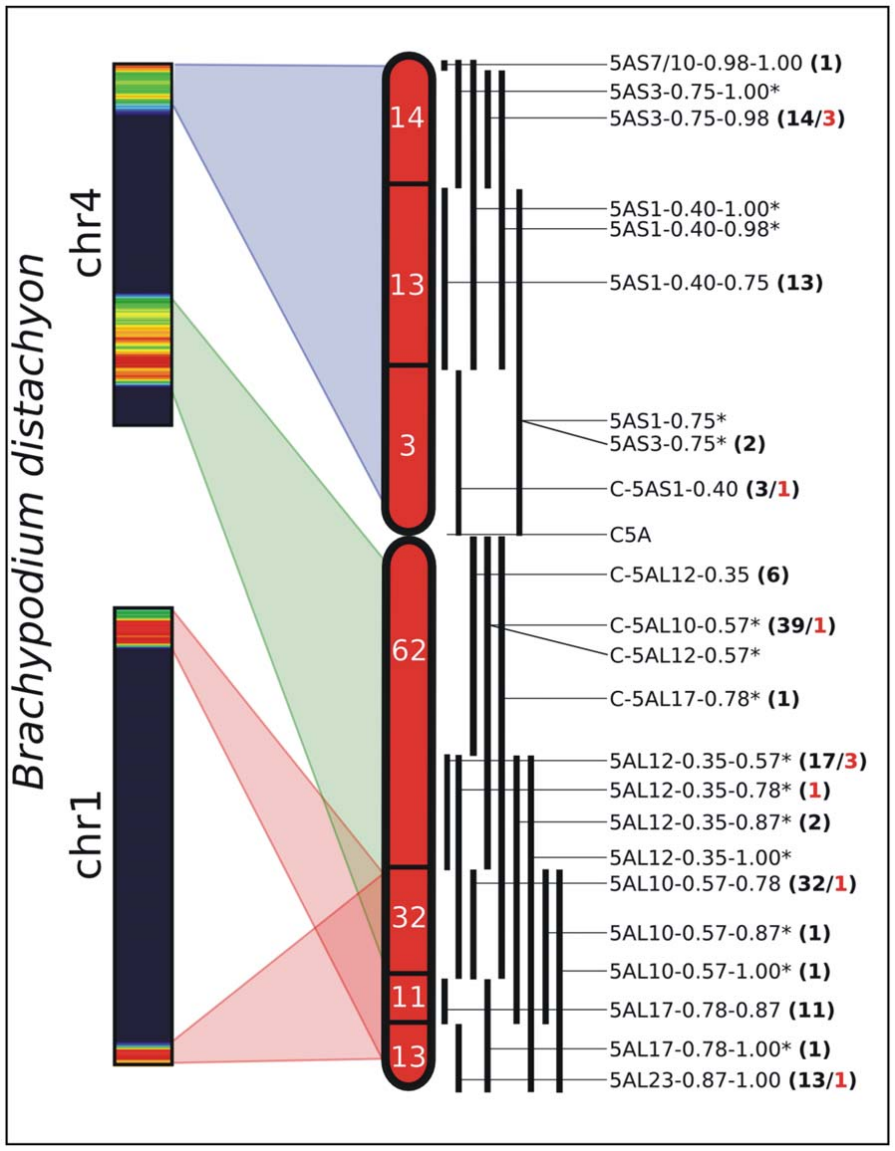
Integration between wheat chromosome 5A virtual gene order and its physical map. The integration was achieved by anchoring the wheat genes positioned along the genome zipper with the EST markers. The figure shows the *Brachypodium* syntenic regions. The white numbers on the wheat chromosome represent the number of EST markers anchored to the corresponding bin (on the right) showing homology with the zipper genes. In case of overlapping bins the numbers were summed. The black numbers between parentheses beside each bin name indicate how many ESTs are anchored on that specific bin. Red numbers indicate genes anchored on the wrong arm with respect to the origin of the reads.

The wheat virtual gene order based on the syntenic regions of *Brachypodium*, rice and sorghum was integrated with the EST markers from and a deletion line-based physical map of chromosome 5A available at the GrainGenes database (http://wheat.pw.usda.gov/GG2/index.shtml). A similarity search of the whole wheat chromosome 5A sequence dataset against the EST markers identified 2,737 and 4,176 reads respectively for 5AS and 5AL arms. 80% and 90% of the reads from the 5AS and 5AL arms, respectively, find a match with an EST marker anchored on the correct arm, while the rest maps on the opposite arm. This phenomenon could be due to presence of a small portion of the opposite arm pericentromeric region, or to errors in marker anchoring. The integration of the genome zipper with the physical map is shown in Figure 6. 47 ESTs map on the 5AS zipper and 139 ESTs on the 5AL zipper. The complete list of zipper genes, for both the short and long arms, is available as supporting information (Table S2).

**Figure 7:**
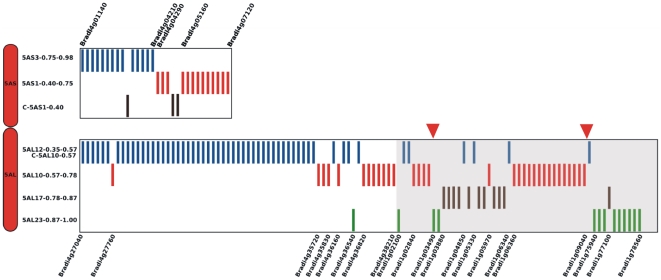
Consistency between virtual gene order and marker distribution. The figure integrates the genome zipper and the deletion bin map (from Figure 6) in order to verify their collinearity. Each gene shared by the two maps is represented by a bar and it is positioned according to the bin (seven levels on the vertical axis, indicated on the left) and to the zipper (horizontal axis). If the two maps are fully consistent then the zipper genes would fill one bin at a time from the top to the bottom of the figure. Although, as expected, the correspondence of the two maps is not perfect, genes within a bin tend to be contiguous within the zipper. The darker area on the 5AL arm represents the portion of the zipper syntenic with *Brachypodium* chromosome 1, while the rest corresponds to *Brachypodium* chromosome 4. The apparent lack of collinearity at the end of the long arm may be explained by an inversion (bracketed by red arrowheads) occurred between wheat and *Brachypodium*. Some relevant zipper genes are indicated at the top and bottom of the figure.

To evaluate the quality of the genome zipper, the consistency between the virtual gene order and the distribution of the EST markers was verified. The analysis was performed considering only non-overlapping bins with high numbers of mapped ESTs, as described in Materials and Methods. Short arm bins contained 30 mapped ESTs, and long arm bins 112 mapped ESTs. The results (Figure 7) show that the order of 5AS and 5AL zipper genes from *Brachypodium* chromosome 4 is coherent with the physical map, as the genes and bin positions follow a very similar order. Indeed only 3 EST markers from 5AS and 6 from 5AL are positioned on non contiguous positions. In contrast, genes from 5AL located on *Brachypodium* chromosome 1 show a less conserved distribution suggesting a probable inversion in wheat (Figure 7) compared to *Brachypodium*.

### Identification of candidate conserved microRNAs

The pipeline described in Materials and Methods allowed the identification of 195 candidate miRNA precursors, 92 and 103 for the short and long arm, respectively. Problems related to the computational identification of false positive miRNA are well known and several strategies have been developed to deal with that. Moreover, because of its polyploid genome, wheat has potentially a lot of different genomic locations coding for miRNAs of the same family, although many of them could be silenced or expressed only in specific conditions. However, among the identified miRNA precursors, 54 were found to be highly similar to precursors already known in plants, while the others can be either false positives or novel precursors. After searching in the Uniprot database, no similarities were found with known proteins for any of the identified sequences. Sixteen miRNA families were shown to be putatively present on chromosome 5A: two of them (miR164 and miR167) were found only in the short arm, three families (miR156, miR399 and miR2118) were found only in the long arm, while the remaining eleven families were found in both arms (Table 3).

**Table 3:** Putative miRNA families identified in the 454 reads of the short and long arms of chromosome 5A.

miRNA family	Homologous miRNAs	Number of precursors	Precursor length	Mature position
**5A short arm**				
164	*O.sativa, S.bicolor*	1	254	5′
167	*B.napus*	1	101	5′
437	*O.sativa, S.bicolor*	1	199	3′
441	*T.aestivum*	4	97–280	3′ - 5′
818	*O.sativa*	6	87–109	3′
1117	*T.aestivum*	5	122–124	3′
1118	*T.aestivum*	6	251–281	3′
1120	*T.aestivum*	20	68–99	3′
1122	*T.aestivum, B.distachyon*	40	75–160	3′ - 5′
1125	*T.aestivum*	1	137	5′
1130	*T.aestivum*	2	96–104	5′
1131	*T.aestivum*	4	81	5′
1847	*O.sativa*	1	83	3′
**5A long arm**				
156	*O.sativa, S.bicolor, S.officinarum, Z.mays*	1	138	5′
399	*O.sativa, A.thaliana, M.truncatula, P.vulgaris, S.bicolor, V.vinifera, Z.mays*	2	93–116	3′
437	*O.sativa, S.bicolor, B.distachyon*	2	194–201	3′
441	*T.aestivum*	3	112–286	3′ - 5′
818	*O.sativa*	14	93–184	3′
1117	*T.aestivum*	8	81–124	3′ - 5′
1118	*T.aestivum*	2	256–259	3′
1120	*T.aestivum*	22	69–249	3′
1122	*T.aestivum, B.distachyon*	39	77–161	3′ - 5′
1125	*T.aestivum*	3	137–138	5′
1130	*T.aestivum*	2	101	5′
1131	*T.aestivum*	3	81	5′
1847	*O.sativa*	1	84	3′
2118	*Z.mays*	1	457	5′

For each family, the table reports the plant species to which the homologous mature microRNAs belong, the number of candidate precursors, their length and the position of the mature sequence in the hairpin.

It is well known that the size of hairpin precursors is more variable in plants than in animals and secondary structures appear to be more complex in the former [37]. Referring to the short arm of 5A, miRNA putative precursors showed an average length of 114.13 nt, with a minimum of 68 nt and a maximum of 281 nt; the average minimum free energy index (MFEI) was 1.47, while the average GC content was 38.19%. In the long arm, miRNA precursors showed an average length of 113.32 nt, with a minimum of 69 and a maximum of 457 (although this value is related to one case that is exceptional compared to the other precursors). The average MFEI was 1.47, while the average GC content was 38.63%. The percentage of mature sequences with U in the first position was 59% in the short arm and 54% in the long arm. These values are in good agreement with those published in previous reports [21]. Moreover, most precursors have the mature sequence in the 3′ end of the hairpin (68% in the short arm, 63% in the long arm). This characteristic feature of wheat miRNAs has already been highlighted by Han et al. [21].

Since the wheat genome is rich in repetitive sequences, the number of candidate precursors containing repeats was calculated. In the short arm, Repeat Masker identified 35 miRNA precursors with at least one known repetitive element (38% of the total number of precursors), while 28 precursors were found in the long arm (27%). The repetitive sequences were all classified as originating from transposable elements. This evidence supports the model proposed by Piriyapongsa and Jordan [38] on how miRNAs could have evolved from TE encoding siRNA.

Some of the miRNA families found on chromosome 5A were associated with a set of predicted or experimentally confirmed targets, involved in developmental or metabolic processes and in stress response.

### Wheat unigenes represented in the 5A snapshot contigs and their use for scaffolding

Wheat unigenes from NCBI (*Triticum aestivum* unigenes v55, containing 40,349 unigenes) were blasted against the contigs (identity ≥97%, L ≥50 bp). The use of the contigs (regions covered by more than one read) and the high stringency applied, should avoid scaffolding genes from homeologous chromosomes present as contaminants in the chromosomal preparation. The following parameters were measured: the number of unigenes matching the contigs, percent coverage of each of these unigenes and the number of contigs matching each unigene. The contigs were matched by 2,772 unigenes, i.e. 6.9% of the total. 634 unigenes matched only 5AS, 1,962 only 5AL and 176 both arms (Table 4). The latter may represent genes located in pericentrometric regions, which, as discussed above, could be present in both flow-sorted chromosome arm fractions if the 5AS and 5AL telosomes were not telocentric but actually acrocentric. The fraction of unigenes mapped compares well with the portion of the wheat genome covered by the chromosome 5A (approx. 4.9% [1]) and is different from the 5,088 genes estimated in the previous sections. The discrepancy is most likely due to the exclusion of the singletons in the present analysis and to the fact that the 40,349 unigenes present in the NCBI unigene build are clearly an underestimate with respect to the projected number of wheat genes, since many homeologous transcripts have been collapsed into a single unigene during construction of the build. The majority of the unigenes recognized only 1–4 contigs, thus representing non-repetitive transcripts. Twelve unigenes contained highly repetitive sequences, judged by the fact that the same region of each unigene was present on >10 distinct contigs with high identity (>97%). Sixteen unigenes showed high sequence similarity to other unigenes in the collection, and recognized the same contigs with different identities; these were assumed to represent homeologs, and only the unigene showing highest sequence similarity with the contigs was retained for scaffolding (see below). In one case, a single unigene (gnl|UG|Ta#S15879726) recognized 25 different contigs, some of them overlapping, that covered its whole sequence multiple times; BlastX analysis showed partial homology with a gag polyprotein, indicating that this unigene derived from a presumed retrotransposon. The detailed list of unigenes mapped is reported in Table S3.

**Table 4:** Unigenes mapped on 5A chromosome arms.

	*N*	*%*
**Total unigenes**	40,349	100
**Unigenes matching 5A**	2,772	6.9
**5A 1 hit**	1,765	4.4
**5A >1 hit**	1,007	2.5
**Only 5AS**	634	1.6
**Only 5AL**	1,962	4.9
**Both arms**	176	0.4

**Figure 8:**
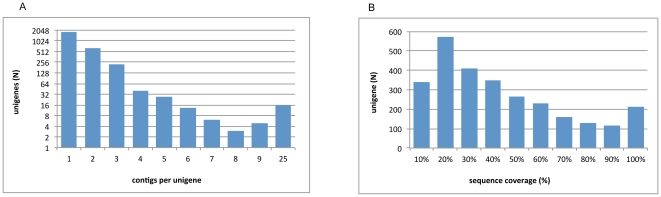
Unigene representation on chromosome 5A assembly. **A**: number of contigs per unigene; **B**: percentage of sequence coverage by unigenes.

**Figure 9:**
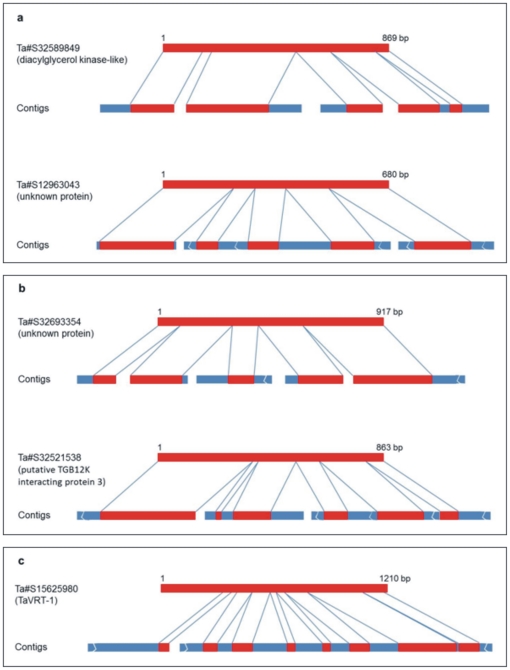
Representative examples of contig scaffolding. **A**: examples of unigenes from 5AS; **B**: examples of unigenes from 5AL; **C**: scaffolding and coverage for gene *TaVRT-1*. Red bars: exon sequences, blue bars: introns or other sequences not present in the mRNA.

The unigenes matching the contigs showed sequence coverage close to 40% (Figure 8). Such low coverage is due to the use of contigs (regions covered by more than one read). A number of unigenes recognized multiple contigs, and each separate contig recognized a different portion of the unigene. These unigenes were used for scaffolding of the contigs with the limitation that the gap length between two joined contigs could not always be determined, due to the presence of introns of unknown length. We were able to produce 207 scaffolds on the short arm, derived from 510 contigs, and 579 scaffolds on the long arm, derived from 1,424 contigs. The average bases increased from approx. 400bp in the contigs, to approx. 2Kb in scaffolds, excluding Ns introduced during the scaffolding procedure (Table 5). These numbers were similar for the short and long arms. Several contigs could be scaffolded to cover a complete unigene (Figure 9).

**Table 5:** Scaffolding statistics. Bases per scaffold are reported, omitting the Ns added to join the contigs.

	5AS	5AL
**N contigs**	159,243	276,222
**N bases per contig**	383	421
**N contigs in unigenes**	510	1,424
**N exons per contig**	1.3	1.3
**N scaffolds**	207	579
**N contigs per scaffold**	2.5	2.5
**N bases per scaffold**	1,988.4	1,782.6
**N exons per scaffold**	3.2	3.1

To evaluate the degree of completeness of the assembly for the non-repetitive gene fraction, the presence of genes already reported to map on chromosome 5A was checked. The following sequences were considered: the vernalization locus *TaVRT-1* [22], the free threshing locus *Q* [26] and the frost tolerance locus *Cbf* comprising of at least 5 genes mapped on 5AL [23]. Of these, portions of *TaVRT-1* (Figure 9) and *Cbf3d*-*A15* matched contigs, whereas other *Cbf* and *Q* genes, with the exception of *Cbf4b-A20*, were only identified in the non-assembled reads (Table S4). All genes mapped on the long arm of 5A, in accordance with previous reports. *Cbf* is a large gene family of transcription factors containing approximately 50 members spread on at least 4–5 different chromosomes [23]; of these, 5 have been mapped on chromosome 5AL, but it is conceivable that the number is higher. A blast-based search conducted using more relaxed parameters (identity ≥80%, L ≥50 bp) identified a number of contigs and singletons, possibly representing other members of the same family.

### Conclusions

The low coverage sequence of wheat chromosome 5A has provided for the first time a description of the main sequence features of this chromosome and new information on transposable elements and their significant contribution to genome size. The information on expressed genes acquired with the low coverage sequencing was used to produce a virtual gene order of chromosome 5A based on its synteny with other phylogenetically-related species. Gaining an early insight on the 5A sequence provides relevant information for finishing the genome assembly and for the isolation of 5A genes controlling important agronomic traits (e.g. Fusarium head blight [25]).

## Materials and Methods

### Flow cytometric chromosome sorting and amplification of chromosomal DNA

The seeds of double ditelosomic line 5A of *Triticum aesticum* cv. Chinese Spring (20″+t″5AS+t″5AL) were provided by Prof. Bikram S. Gill (Kansas State University Manhattan, USA). The dDt5A line was originally developed by Sears [39]. Liquid suspensions of intact mitotic chromosomes were prepared from synchronized root tip meristems of young seedlings according to Vrána et al. [40] and Kubaláková et al. [41]. The suspensions were stained with 2 µg/ml DAPI (4′,6-diamino-2-phenylindole) and 33,000 and 19,000 copies of telocentric chromosomes 5AS and 5AL, respectively, were sorted using a FACSVantage SE flow cytometer (Becton Dickinson, San José, USA) into 0.5ml microtubes containing 40 µl sterile deionized water. Five independent samples were prepared from 5AS and two samples were prepared from 5AL. The identity of sorted chromosomes and presence of contaminating chromosomes was done using fluorescence *in situ* hybridization (FISH) according to Kubaláková et al. [42] using probes for telomeric repeat for both arms, pSc119.2 repeat [43] for 5AS and GAA microsatellite and/or Afa-repeat [44] for 5AL (Figure S1). DNA of flow-sorted telocentrics was amplified as described in Šimková et al. [27]. Briefly, the chromosomes were treated with proteinase K and after its removal, chromosomal DNA was amplified by the illustra GenomiPhi V2 DNA Amplification Kit (GE Healthcare Bio-Sciences Corp., Piscataway, USA) in a 20-µl reaction volume. The concentration of the amplification product was estimated fluorometrically using a Modulus Single Tube Multimode Reader (Turner Biosystems, Sunnyvale, USA).

### 454 sequencing and assembly

454 GS-FLX Titanium sequencing was used to generate fragment reads from short and long wheat chromosome 5A arms. Both libraries have been prepared and sequenced independently. Long-arm 5A chromosome DNA 454 libraries were amplified by Large Volume emulsion PCR, using 7 molecules of DNA per bead, following the supplier's instructions (http://www.roche.com). After the amplification step, the emulsion was broken chemically and the beads carrying the amplified DNA library were recovered and washed; DNA bead-immobilized templates were then denatured and the sequencing primer was annealed (GS FLX Titanium emPCR Method Manual). The DNA samples, clonally amplified and bead-immobilized, were sequenced by the 454 GS FLX Titanium sequencer, using 70x75 picotiter plates, as described in the GS FLX Titanium Sequencing Method Manual (Roche).

Assembly was performed with Newbler v 2.3 (ROCHE) using the “large genome” option, useful for complex genomes rich in repeated regions and to speed up computational time.

The 454 reads obtained in this work were deposited in the NCBI-Sequence Read Archive database (http://www.ncbi.nlm.nih.gov/sra) under the accession numbers SRX031791 (5A short arm) and SRX031790 (5A long arm); while the assembled contigs were deposited in the NCBI-Whole Genome Shotgun database (http://www.ncbi.nlm.nih.gov/genomeprj) under the ProjectID 15528 that represents an umbrella project for the whole International Wheat Genome Sequencing Consortium's project, the subproject for 5A chromosome has the accession n. 61773.

### Characterization of repeat fraction

Blast analyses for repeat characterization were conducted locally on 454 reads using a cutoff Expect value of 10e^−6^ [31]. The reference databases used were downloaded from Triticeae database (TREP complete and PTREP databases, http://wheat.pw.usda.gov/ITMI/Repeats). Blast analyses were driven by custom Python scripts allowing recovery of non-matching reads and immediate parsing of the BLAST output to limit file size. Various related functions as ID-based retrieval of complete fasta records from 454-raw output fasta files where implemented with memory-efficient BioPython-based modules [45] by in-house Python scripts.

In order to identify as many repetitive elements as possible, two additional filtering steps were performed. First, a similarity search against virus proteins downloaded from NCBI (ftp://ftp.ncbi.nih.gov/genomes/Viruses/) was run. The 454 reads were aligned against this dataset using BlastX and the reads were classified as “viral proteins” when the following parameters were satisfied: E-value ≥0,001; Identity ≥30; Similarity ≥50; Reads coverage ≥50%; protein coverage ≥1%. In a second filtering step, all reads matching against assembled contigs with a coverage ≥5x were excluded. This threshold was set taking into consideration that the average sequencing coverage was estimated in 2.3x.

The “Repeats” class was defined as containing reads escaping previous TE and gene classes. High coverage contigs were estimated as those above a cutoff of 5 according to the following formula: (nr)/c, where n represents the number of reads participating to a contig, r is the average read length and c is the length of the contig consensus sequence. Due to the low-coverage snapshot, high coverage contigs are very likely to represent *sensu stricto* repeats [12].

### Gene Content analysis

The 454 reads filtered by TEs and repetitive regions were used to perform a similarity search both at the protein and nucleotide level against the following databases:

*a) Monocots Uniprot* (Swissprot and TREMBL release 2010_05 - http://www.uniprot.org/): all Uniprot entries belonging to monocotyledon species according to NCBI Taxonomy were selected and the similarity search was run by BlastX considering as positive matches the hits with E-value ≤e^−6^, Identity ≥30%; Similarity ≥50%; Read coverage ≥50%; Database entry coverage ≥1%.

*b) Liliopsida clustered Unigene* (http://www.ncbi.nlm.nih.gov/unigene): this unigene dataset contains sequences from *Hordeum vulgare*, *Oryza sativa*, *Panicum virgatum*, *Saccharum officinarum*, *Sorghum bicolor*, *Triticum aesitvum* and *Zea mays* species. A similarity search was performed using BlastN and significant matches were assessed using the same parameters as in Uniprot analysis, with the exception of E-value cut-off that was set to 10e^−30^.

Despite the filters used to identify repetitive elements, some reads matching with proteins annotated by PFAM as transposon, retrotransposon and integrase were found. For these reasons all sequences matching with proteins with the following Pfam code were excluded: PF00078, PF03732, PF05380, PF07727, PF07999,PF08284, PF12382, PF00098, PF02992, PF03004, PF03017, PF04827, PF10536, PF10551, PF12620, PF01021, PF04195, PF01480, PF04094, PF05754, PF00665.

### Syntenic regions analysis

The *Brachypodium* proteome was downloaded from http://www.brachypodium.org, rice proteome from ftp://ftp.ncbi.nih.gov/genomes/Oryza_sativa/ and sorghum proteome from http://www.phytozome.net. The 454 reads filtered by TEs and repetitive regions were used to perform a BlastX similarity search considering as positive only the best hits with E-value ≤e^−6^, Identity ≥30%; Similarity ≥50%; Read coverage ≥50%. Taking into consideration the parameters used and the low coverage of the genomic sequence, we cannot exclude the presence of false positive matches due to pseudogenes, paralogue genes or other ambiguous gene fragments that would produce background noise genes. We try to minimize this problem considering only the best hit.

### Chromosome zipper

To create the zippers only the *Brachypodium* genes showing homology at DNA level with either rice or sorghum or both and with a similarity E-value lower that 10e^−10^ were used. The chromosome zipper was produced as described by Mayer at al. [6], then, the orthologous relationships among the genes were verified using the “best reciprocal hit” method. Each gene of the zipper was described with a code which gives an indication of the robustness of the predicted syntenic region. The code has the following syntax OxSx(hyz), where O and S means *Oryza* and *Sorghum* respectively, while x, h, y and z can take the binary value of 1 or 0. The first part of the code, OxSx, indicates if the homologous genes from rice and sorghum are located on the syntenic regions, setting the value of “x” to 1 if the condition is true, to 0 otherwise. The second part of the code, (hyz), is set to 1 if the genes are true orthologous as verified using the best reciprocal hit, otherwise to 0. The three digits refer to the relationship between *Brachypodium*-rice (h), *Brachypodium*-sorghum (y) and rice-sorghum (z).

The regions syntenic to wheat chromosome 5A are located on the *Brachypodium* chromosome 4 and chromosome 1. The chromosome 5AS corresponds to a region on chromosome 4 between the genes Bradi4g00210 and Bradi4g08150, while the 5AL syntenic region corresponds to *Brachypodium* chromosome 4 between the genes Bradi4g26960 and Bradi4g39000. The end of chromosome 5AL is syntenic to *Brachypodium* chromosome 1 between the genes Bradi1g00200 and Bradi1g09130 and between Bradi1g74380 and Bradi1g78830. The EST markers were downloaded from GrainGenes web site (http://wheat.pw.usda.gov/GG2/index.shtml). Wheat 454 reads showing a similarity with an ESTs with an E-value lower than 10e^−10^ were anchored to the corresponding bin.

Comparison analysis between ESTs and zipper genes distribution has been performed considering only those non overlapping been with the higher number og mapped est. We selected short arm bin 5AS3-0.75–0.98, 5AS1-0.40–0.75 and C-5AS1–0.40 and long arm bin 5AL12-0.35–0.57 and C-5AL10-0.57, 5AL10-0.57–0.78, 5AL17-0.78–0.87, 5AL23-0.87–1.00.

### microRNA identification and analysis

Candidate miRNA related sequences were identified in the 454 sequences of wheat chromosome 5A, according to the following pipeline: i) BlastN search of known miRNA mature sequences, ii) Data filtering, iii) Precursor extraction and iv), Prediction of secondary structure.

- All the known plant mature miRNAs stored in the miRBase (release 14) were used as query sequences to perform a BlastN search against the contigs and the singletons of both arms of the chromosome 5A. Due to the huge quantity of data, slightly more stringent parameters were used compared to those published previously [46]: e-value 10, maximum number of hits 10 and word size 7.
- All the Blast hits were split into two groups: direct hits and reverse complement hits; direct hits with more than 3 mismatches with the known sequence were discarded [47], while all the reverse complement hits were retained. Only the singletons and the contigs with both a direct and a reverse complement hit were retained for further analysis since true miRNA precursor should have both a mature sequence on one arm of the hairpin and a paired passenger sequence (called miRNA*) on the opposite arm; moreover, while the mature sequence is known to be highly conserved, the passenger sequence could be more variable. This strategy was useful to exclude sequences which are less likely to fold with a hairpin structure.
- miRNA precursors were extracted from the sequences by cutting 13 nt before the 5′ hit and 13 nt after the 3′ hit, since this region (from end of miRNA to end of precursor - pri-extension region of the hairpin) was recently shown to have this average length in plants [48].
- The secondary structure of the precursors was predicted with the software mFold 3.2 [49]. The minimal folding free energy index (MFEI) was calculated for each structure by the equation:

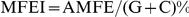



where AMFE (Adjusted MFE) is the minimal free energy of 100 nucleotides. All the sequences with a MFEI greater than 0.85 were considered potential miRNA precursors [50]; besides, only 4 mismatches were allowed between the mature sequence and the passenger sequence, and only few and small asymmetric bulges were accepted [51]. All the precursors were then blasted against the Uniprot database filtered for monocot proteins to exclude those that belonged to protein-coding sequences; the thresholds used were E-value <0.001, Identity >30%, Similarity >50%, read coverage >50%, Protein coverage >1%. The candidate precursors were blasted against known plant microRNAs (miRBase 16), and were considered similar to a known precursor if they had an identity percent greater than 70. Lastly, the identified miRNAs were analyzed for the presence of repetitive sequences by using the Repeat Masker Web Server (http://www.repeatmasker.org) with default settings.

### Verification of unigene coverage by the assembly and Unigene-based scaffolding

The wheat unigenes were downloaded from GenBank (*Triticum aestivum* unigenes, v55, updated Feb 2009, http://www.ncbi.nlm.nih.gov/UniGene/UGOrg.cgi?TAXID=4565) without further modifications and were used to assess the coverage provided by the 5AS and 5AL assemblies. Additionally, they were checked against the Triticeae Repeat Sequence Database (TREP, updated July 2008) for repetitive sequences that may lead to unspecific mapping.

Sequence similarity analyses were assessed by BlastN; a positive score was assigned to those sequences matching the databases with an identity ≥97%. The coverage length threshold could not be quantitatively determined since RNA-derived sequences were used, 50bp windows were considered as minimum overlap. The coverage of the unigenes by the contigs of the assemblies was calculated with a script developed in-house based on Blast-derived data.

The Unigenes uniquely and univocally matching more than one contig were used to perform scaffolding of the contigs generated by the Newbler assembler; this was done to decrease the number of contigs and to obtain scaffolds representing actively transcribed genomic sequences. The analyses were performed by implementing a program in C language, based on the data obtained by the sequence similarity analyses from above. Since the effective span between contigs connected by this scaffolding method could not be assessed, they were arbitrarily joined with a row of 100 Ns.

## Supporting Information

Figure S1 Flow karyotype of double ditelosomic line Chinese Spring dDt5A. The karyotype consists of three composite peaks (I – III) representing groups of chromosomes, a peak representing chromosome 3B, and peaks representing chromosome arms 5AS and 5AL. X axis: relative DAPI fluorescence intensity; Y axis: number of events. The inserts show examples of flow-sorted chromosomes 5AS and 5AL after FISH with DNA probes. Chromosome 5AS was identified with probes for telomeric repeat (green signals) and pSc119.2 (red signals). Chromosome 5AL was identified with probes for telomeric repeat (red signals) GAA microsatellite (green signals). The chromosomes were counterstained with DAPI (blue color).
(DOC)

Table S1 Complete list of wheat 5A Transposon Element families abundance.
(DOC)

Table S2 Complete list of *Brachypodium* genes homologous to the wheat 5A sequences ordered according to the position in the *Brachypodium* genome.
(XLSX)

Table S3 Complete list Unigenes mapped on 5A.
(XLSX)

Table S4 Known genes of chromosome 5A mapped on the assembly.
(XLSX)
