# Supplementary material for: First Survey of the Wheat Chromosome 5A Composition through a Next Generation Sequencing Approach
Source: PLoS One. 2011 Oct 18;6(10):e26421. doi: 10.1371/journal.pone.0026421 (PMC3196578; doi:10.1371/journal.pone.0026421)
Supplement: Figure S1 — Flow karyotype of double ditelosomic line Chinese Spring dDt5A. The karyotype consists of three composite peaks (I – III) representing groups of chromosomes, a peak representing chromosome 3B, and peaks representing chromosome arms 5AS and 5AL. X axis: relative DAPI fluorescence intensity; Y axis: number of events. The inserts show examples of flow-sorted chromosomes 5AS and 5AL after FISH with DNA probes. Chromosome 5AS was identified with probes for telomeric repeat (green signals) and pSc119.2 (red signals). Chromosome 5AL was identified with probes for telomeric repeat (red signals) GAA microsatellite (green signals). The chromosomes were counterstained with DAPI (blue color). (DOC) [file pone.0026421.s001.doc]

**First Survey of the Wheat Chromosome 5A Composition through a Next Generation Sequencing Approach (Vitulo et al)**

**
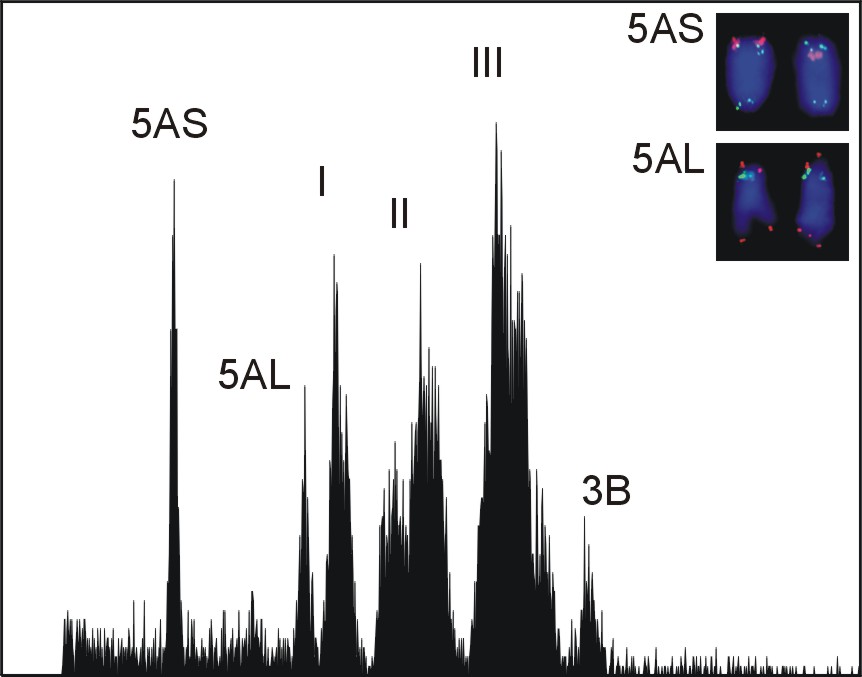
**
