# Supplementary material for: First Survey of the Wheat Chromosome 5A Composition through a Next Generation Sequencing Approach
Source: PLoS One. 2011 Oct 18;6(10):e26421. doi: 10.1371/journal.pone.0026421 (PMC3196578; doi:10.1371/journal.pone.0026421)
Supplement: Table S1 — Complete list of wheat 5A Transposon Element families abundance. (DOC) [file pone.0026421.s002.doc]

**First Survey of the Wheat Chromosome 5A Composition through a Next Generation Sequencing Approach (Vitulo et al)**

**Supplementary Table 1: complete list of wheat 5A Transposon Element families and apparent** abundance

| **TE_family (or subfamily)** | **long arm reads** | **% long arm** | **short arm reads** | **% short arm** |
| --- | --- | --- | --- | --- |
| RLG_Sabrina | 497,607 | 18.203192 | 339,579 | 18.524808 |
| RLG_WHAM | 224,746 | 8.221537 | 153,417 | 8.369247 |
| RLG_Fatima | 173,557 | 6.348969 | 113,647 | 6.199703 |
| RLG_Wilma | 159,792 | 5.845425 | 102,670 | 5.600882 |
| RLC_Angela | 153,516 | 5.615840 | 84,451 | 4.606994 |
| DTC_Jorge | 130,451 | 4.772088 | 111,700 | 6.093490 |
| RLG_Egug | 68,393 | 2.501916 | 49,623 | 2.707048 |
| RLC_WIS | 61,095 | 2.234944 | 28,030 | 1.529100 |
| RLC_Barbara | 59,202 | 2.165696 | 35,321 | 1.926841 |
| RLG_Erika | 53,661 | 1.962998 | 65,816 | 3.590413 |
| RLG_Laura | 51,967 | 1.901029 | 41,155 | 2.245099 |
| RLC_Maximus | 50,080 | 1.832000 | 32,577 | 1.777150 |
| RLG_Sakura | 49,867 | 1.824208 | 31,245 | 1.704486 |
| RLG_Romani | 44,990 | 1.645800 | 37,483 | 2.044783 |
| RLG_Derami | 41,811 | 1.529508 | 27,707 | 1.511480 |
| RLG_Cereba | 38,012 | 1.390535 | 122,878 | 6.703275 |
| RLG_Ifis | 25,648 | 0.938241 | 15,435 | 0.842014 |
| RLC_Claudia | 25,250 | 0.923682 | 17,658 | 0.963284 |
| DTC_Clifford | 23,596 | 0.863176 | 11,417 | 0.622823 |
| RLG_Jeli | 22,933 | 0.838923 | 14,794 | 0.807046 |
| RLG_Sumana | 21,641 | 0.791659 | 20,574 | 1.122359 |
| RLG_Nusif | 21,064 | 0.770552 | 19,510 | 1.064315 |
| RLG_Carmilla | 20,996 | 0.768064 | 12,060 | 0.657900 |
| DTC_Pavel | 19,850 | 0.726142 | 21,315 | 1.162782 |
| RLG_Sumaya | 17,766 | 0.649906 | 17,999 | 0.981886 |
| DTC_TAT1 | 13,994 | 0.511921 | 9,024 | 0.492280 |
| RLG_Latidu | 13,458 | 0.492313 | 10,500 | 0.572799 |
| RLC_Inga | 11,228 | 0.410737 | 5,314 | 0.289891 |
| DTC_Caspar | 11,079 | 0.405286 | 4,587 | 0.250231 |
| RLX_Gujog | 9,582 | 0.350524 | 6,578 | 0.358845 |
| RLG_Romana | 9,138 | 0.334281 | 8,232 | 0.449074 |
| RLC_Eugene | 8,562 | 0.313210 | 4,629 | 0.252522 |
| DTT_Thalos | 7,133 | 0.260936 | 3,534 | 0.192788 |
| RLG_BAGY2 | 7,077 | 0.258887 | 5,316 | 0.290000 |
| RLG_Lila | 7,032 | 0.257241 | 5,133 | 0.280017 |
| DTC_Conan | 6,960 | 0.254607 | 2,372 | 0.129398 |
| RLX_Ginger | 6,709 | 0.245425 | 4,563 | 0.248922 |
| RLX_Xalax | 6,698 | 0.245023 | 5,271 | 0.287545 |
| DTC_Vincent | 6,571 | 0.240377 | 3,377 | 0.184223 |
| RLG_Danae | 6,488 | 0.237341 | 7,345 | 0.400686 |
| RLG_Hawi | 6,043 | 0.221062 | 4,333 | 0.236375 |
| DTC_Balduin | 5,976 | 0.218611 | 5,084 | 0.277344 |
| DTC_Byron | 5,837 | 0.213526 | 2,440 | 0.133108 |
| XXX_unnamed | 5,631 | 0.205990 | 15,308 | 0.835086 |
| DTC_Boris | 5,482 | 0.200540 | 3,580 | 0.195297 |
| RLG_Quinta | 5,475 | 0.200284 | 23,126 | 1.261576 |
| DTM_Deimos | 5,329 | 0.194943 | 4,784 | 0.260978 |
| RIX_Karin | 4,932 | 0.180420 | 2,689 | 0.146691 |
| RLG_Surya | 4,489 | 0.164214 | 4,720 | 0.257487 |
| DTT_Icarus | 4,326 | 0.158251 | 2,083 | 0.113632 |
| DTT_Athos | 4,081 | 0.149289 | 1,586 | 0.086520 |
| RLG_Daniela | 3,990 | 0.145960 | 2,322 | 0.126670 |
| DTC_Isaac | 3,636 | 0.133010 | 1,103 | 0.060171 |
| DTC_Jude | 3,390 | 0.124011 | 3,705 | 0.202116 |
| DTC_Hamlet | 3,374 | 0.123426 | 1,404 | 0.076591 |
| RLC_TAR2 | 3,313 | 0.121194 | 1,328 | 0.072445 |
| DTC_Donald | 3,180 | 0.116329 | 1,478 | 0.080628 |
| RLC_Ale | 2,837 | 0.103782 | 1,566 | 0.085429 |
| RIX_Stasy | 2,532 | 0.092624 | 1,272 | 0.069390 |
| DTC_Mandrake | 2,327 | 0.085125 | 1,219 | 0.066499 |
| RLG_Jela | 2,258 | 0.082601 | 1,308 | 0.071354 |
| DTC_TAT2 | 2,218 | 0.081138 | 1,305 | 0.071191 |
| XXX_XC | 2,206 | 0.080699 | 617 | 0.033659 |
| DTT_Stolos | 2,204 | 0.080626 | 986 | 0.053789 |
| DTC_Manor | 2,149 | 0.078614 | 1184 | 0.064590 |
| RLC_Sasanda | 2,128 | 0.077845 | 805 | 0.043915 |
| RLC_unnamed | 2,120 | 0.077553 | 1160 | 0.063281 |
| DTC_Heyjude | 2,044 | 0.074773 | 911 | 0.049697 |
| DTT_Polyphemus | 2,018 | 0.073821 | 1058 | 0.057716 |
| RLC_BARE1 | 1,911 | 0.069907 | 1558 | 0.084992 |
| DTT_Hades | 1,888 | 0.069066 | 737 | 0.040205 |
| RLC_Usier | 1,676 | 0.061311 | 1205 | 0.065735 |
| DTH_Kong | 1,576 | 0.057652 | 699 | 0.038132 |
| DTC_Sherlock | 1,551 | 0.056738 | 1013 | 0.055261 |
| RLC_TAR1 | 1,526 | 0.055823 | 809 | 0.044133 |
| DTC_unnamed | 1,497 | 0.054762 | 1091 | 0.059517 |
| XXX_Demeter | 1,467 | 0.053665 | 1844 | 0.100594 |
| RLG_Heidi | 1,441 | 0.052714 | 1784 | 0.097321 |
| RLC_Leojyg | 1,377 | 0.050373 | 691 | 0.037696 |
| RLG_Lisa | 1,274 | 0.046605 | 1016 | 0.055425 |
| RLC_Daniela | 1,234 | 0.045142 | 1146 | 0.062517 |
| RLX_Veju | 1,228 | 0.044922 | 486 | 0.026512 |
| DTT_Fortuna | 1,216 | 0.044483 | 1048 | 0.057171 |
| DTC_Fergat | 1,209 | 0.044227 | 731 | 0.039878 |
| DTC_Norman | 1,111 | 0.040642 | 617 | 0.033659 |
| DTM_Remus | 1,087 | 0.039764 | 423 | 0.023076 |
| RIX_Miuse | 1,072 | 0.039215 | 660 | 0.036005 |
| RLG_unnamed | 1,009 | 0.036911 | 573 | 0.031258 |
| DTH_Coeus | 997 | 0.036472 | 469 | 0.025585 |
| DTC_Enac | 980 | 0.035850 | 433 | 0.023621 |
| DTT_Pan | 938 | 0.034313 | 334 | 0.018220 |
| DTH_Orpheus | 926 | 0.033874 | 476 | 0.025967 |
| RLC_Valerie | 900 | 0.032923 | 480 | 0.026185 |
| RIX_unnamed | 897 | 0.032814 | 500 | 0.027276 |
| DTH_Xenon | 895 | 0.032740 | 416 | 0.022694 |
| RLG_Haight | 881 | 0.032228 | 573 | 0.031258 |
| XXX_Walter | 850 | 0.031094 | 413 | 0.022530 |
| DTT_Oleus | 780 | 0.028534 | 323 | 0.017620 |
| RLX_Cassandra | 735 | 0.026887 | 545 | 0.029731 |
| RLC_Bianca | 733 | 0.026814 | 265 | 0.014456 |
| DTH_Rong | 723 | 0.026448 | 304 | 0.016584 |
| DTH_Kerberos | 649 | 0.023741 | 515 | 0.028094 |
| XXX_Xenos | 645 | 0.023595 | 217 | 0.011838 |
| RLG_Olivia | 643 | 0.023522 | 493 | 0.026894 |
| DHH_Helios | 619 | 0.022644 | 323 | 0.017620 |
| XXX_Xumet | 610 | 0.022315 | 396 | 0.021603 |
| DTT_Eos | 607 | 0.022205 | 525 | 0.028640 |
| RLX_Artem | 602 | 0.022022 | 508 | 0.027713 |
| RLC_Oref | 591 | 0.021620 | 334 | 0.018220 |
| RIX_Ramona | 590 | 0.021583 | 189 | 0.010310 |
| XXX_Iapetus | 578 | 0.021144 | 467 | 0.025476 |
| DTT_Tantalos | 577 | 0.021108 | 345 | 0.018821 |
| RLC_Olivia | 562 | 0.020559 | 288 | 0.015711 |
| RIX_Isabelle | 545 | 0.019937 | 229 | 0.012492 |
| RLC_Rada | 535 | 0.019571 | 407 | 0.022203 |
| RLC_Ikya | 517 | 0.018913 | 161 | 0.008783 |
| RLX_Varna | 514 | 0.018803 | 501 | 0.027331 |
| DTT_Mercia | 503 | 0.018400 | 268 | 0.014620 |
| XXX_Emma | 502 | 0.018364 | 138 | 0.007528 |
| DTM_HORMU2 | 500 | 0.018291 | 260 | 0.014184 |
| DTM_Rhea | 474 | 0.017340 | 312 | 0.017020 |
| RLC_Gina | 473 | 0.017303 | 41 | 0.002237 |
| RIX_Yvonne | 470 | 0.017193 | 275 | 0.015002 |
| XXX_Xabor | 469 | 0.017157 | 286 | 0.015602 |
| DTC_TAT5 | 469 | 0.017157 | 302 | 0.016475 |
| XXX_XI | 462 | 0.016901 | 186 | 0.010147 |
| XXX_Xanti | 453 | 0.016571 | 223 | 0.012165 |
| DTH_Victor | 440 | 0.016096 | 232 | 0.012656 |
| DTC_Janus | 440 | 0.016096 | 205 | 0.011183 |
| RLC_Herman | 440 | 0.016096 | 54 | 0.002946 |
| DXX_Xitos | 437 | 0.015986 | 101 | 0.005510 |
| DTM_HORMU | 435 | 0.015913 | 152 | 0.008292 |
| RLC_Giselle | 405 | 0.014815 | 270 | 0.014729 |
| XXX_XIR | 396 | 0.014486 | 86 | 0.004691 |
| XXX_Xobar | 363 | 0.013279 | 202 | 0.011020 |
| DTM_Gerald | 339 | 0.012401 | 133 | 0.007255 |
| DTM_Oscar | 337 | 0.012328 | 40 | 0.002182 |
| DTT_Aison | 336 | 0.012291 | 172 | 0.009383 |
| DTH_unnamed | 321 | 0.011743 | 118 | 0.006437 |
| RLX_Michelle | 310 | 0.011340 | 248 | 0.013529 |
| DTM_Argo | 297 | 0.010865 | 82 | 0.004473 |
| RLG_Geneva | 293 | 0.010718 | 97 | 0.005292 |
| DTH_Islay | 286 | 0.010462 | 224 | 0.012220 |
| RLC_Ida | 275 | 0.010060 | 101 | 0.005510 |
| RIX_Paula | 270 | 0.009877 | 113 | 0.006164 |
| RIX_Morpheus | 266 | 0.009731 | 126 | 0.006874 |
| XXX_Xian | 264 | 0.009658 | 115 | 0.006274 |
| RIX_Reina | 261 | 0.009548 | 100 | 0.005455 |
| DXX_Misenos | 256 | 0.009365 | 105 | 0.005728 |
| DTM_Apollo | 252 | 0.009219 | 86 | 0.004691 |
| RLG_HORGY | 251 | 0.009182 | 186 | 0.010147 |
| XXX_Xi | 250 | 0.009145 | 103 | 0.005619 |
| RLC_Inav | 241 | 0.008816 | 50 | 0.002728 |
| XXX_Xusuf | 240 | 0.008780 | 78 | 0.004255 |
| DTC_Calvin | 231 | 0.008450 | 117 | 0.006383 |
| RLG_Lolaog | 222 | 0.008121 | 143 | 0.007801 |
| DTC_Radon | 222 | 0.008121 | 41 | 0.002237 |
| RIX_Nicole | 216 | 0.007902 | 106 | 0.005783 |
| XXX_XG | 216 | 0.007902 | 176 | 0.009601 |
| RLX_Eway | 214 | 0.007828 | 104 | 0.005673 |
| RLG_Zuhra | 213 | 0.007792 | 227 | 0.012383 |
| DTT_Phoebus | 211 | 0.007719 | 50 | 0.002728 |
| DTC_Nico | 206 | 0.007536 | 91 | 0.004964 |
| DTC_Joey | 206 | 0.007536 | 152 | 0.008292 |
| DTM_Hermes | 198 | 0.007243 | 49 | 0.002673 |
| DTM_Spring | 193 | 0.007060 | 74 | 0.004037 |
| RLC_Ikeros | 190 | 0.006950 | 100 | 0.005455 |
| DTT_Jason | 189 | 0.006914 | 62 | 0.003382 |
| DTM_Argus | 189 | 0.006914 | 130 | 0.007092 |
| RLG_Vagabond | 187 | 0.006841 | 180 | 0.009819 |
| DTC_Storm | 187 | 0.006841 | 55 | 0.003000 |
| DTC_TAT3 | 181 | 0.006621 | 127 | 0.006928 |
| RLC_TAR3 | 180 | 0.006585 | 42 | 0.002291 |
| DXX_George | 175 | 0.006402 | 90 | 0.004910 |
| RIX_Ophelia | 167 | 0.006109 | 66 | 0.003600 |
| RLC_Elena | 165 | 0.006036 | 81 | 0.004419 |
| XXX_Xusag | 162 | 0.005926 | 54 | 0.002946 |
| DTH_OsKong | 162 | 0.005926 | 44 | 0.002400 |
| RLC_Boba | 161 | 0.005890 | 17 | 0.000927 |
| RLC_Lara | 160 | 0.005853 | 106 | 0.005783 |
| RLX_Martin | 160 | 0.005853 | 33 | 0.001800 |
| DTM_Vacuna | 152 | 0.005560 | 49 | 0.002673 |
| DTC_Dagobert | 146 | 0.005341 | 26 | 0.001418 |
| RLC_HORPIA2 | 142 | 0.005195 | 34 | 0.001855 |
| XXX_Xajek | 140 | 0.005121 | 68 | 0.003710 |
| DTC_Korbin | 135 | 0.004938 | 41 | 0.002237 |
| RLG_Sukkula | 129 | 0.004719 | 147 | 0.008019 |
| DTM_Joseph | 124 | 0.004536 | 21 | 0.001146 |
| RIX_Persephone | 110 | 0.004024 | 49 | 0.002673 |
| RLG_BAGY1 | 109 | 0.003987 | 63 | 0.003437 |
| DTT_Pluto | 106 | 0.003878 | 54 | 0.002946 |
| DTT_Antonio | 106 | 0.003878 | 72 | 0.003928 |
| DTC_Gaston | 105 | 0.003841 | 133 | 0.007255 |
| XXX_TaiI | 104 | 0.003804 | 1348 | 0.073536 |
| DTM_Pilifon | 103 | 0.003768 | 34 | 0.001855 |
| DTC_Baldur | 102 | 0.003731 | 54 | 0.002946 |
| DTC_Garth | 92 | 0.003365 | 24 | 0.001309 |
| RLX_Ronda | 83 | 0.003036 | 44 | 0.002400 |
| DTM_Murray | 82 | 0.003000 | 19 | 0.001036 |
| XXX_Milton | 81 | 0.002963 | 33 | 0.001800 |
| DTM_Annie | 80 | 0.002927 | 26 | 0.001418 |
| DTA_Aurec | 79 | 0.002890 | 6 | 0.000327 |
| DTC_TAT4 | 79 | 0.002890 | 67 | 0.003655 |
| DTC_Ivan | 74 | 0.002707 | 30 | 0.001637 |
| DTC_VincentTIR | 73 | 0.002670 | 46 | 0.002509 |
| RLG_Jody | 72 | 0.002634 | 61 | 0.003328 |
| SIX_Dido | 71 | 0.002597 | 32 | 0.001746 |
| DTC_Oswald | 66 | 0.002414 | 24 | 0.001309 |
| XXX_Enuc | 64 | 0.002341 | 27 | 0.001473 |
| DXX_unnamed | 61 | 0.002231 | 14 | 0.000764 |
| DTH_Jura | 60 | 0.002195 | 17 | 0.000927 |
| RLC_Hopscotch | 60 | 0.002195 | 9 | 0.000491 |
| RLX_Xalas | 61 | 0.002231 | 196 | 0.010692 |
| DTM_Zeus | 57 | 0.002085 | 12 | 0.000655 |
| RLC_Liuling | 55 | 0.002012 | 27 | 0.001473 |
| XXX_Nancy | 55 | 0.002012 | 15 | 0.000818 |
| RLC_Zenia | 54 | 0.001975 | 9 | 0.000491 |
| DTM_Troy | 54 | 0.001975 | 7 | 0.000382 |
| XXX_Horus | 51 | 0.001866 | 30 | 0.001637 |
| RLC_Glenda | 51 | 0.001866 | 11 | 0.000600 |
| RIX_Nala | 49 | 0.001792 | 40 | 0.002182 |
| DTC_Kane | 49 | 0.001792 | 19 | 0.001036 |
| RLC_Anna | 47 | 0.001719 | 14 | 0.000764 |
| DTC_Preston | 45 | 0.001646 | 2 | 0.000109 |
| DTC_Aron | 43 | 0.001573 | 15 | 0.000818 |
| RLC_Georgia | 42 | 0.001536 | 9 | 0.000491 |
| DHH_Paddington | 41 | 0.001500 | 18 | 0.000982 |
| RLG_Sabine | 38 | 0.001390 | 13 | 0.000709 |
| XXX_Xevox | 37 | 0.001354 | 17 | 0.000927 |
| RLC_TAR4 | 37 | 0.001354 | 11 | 0.000600 |
| XXX_X1 | 36 | 0.001317 | 10 | 0.000546 |
| DTH_Zong | 35 | 0.001280 | 25 | 0.001364 |
| DTM_Gabriel | 35 | 0.001280 | 5 | 0.000273 |
| RLC_Rita | 33 | 0.001207 | 4 | 0.000218 |
| DXX_Nisos | 33 | 0.001207 | 18 | 0.000982 |
| DTC_Cameron | 31 | 0.001134 | 6 | 0.000327 |
| DTX_Keres | 31 | 0.001134 | 11 | 0.000600 |
| DTC_Sandro | 30 | 0.001097 | 73 | 0.003982 |
| RLC_Hilda | 30 | 0.001097 | 19 | 0.001036 |
| RLC_Ivana | 28 | 0.001024 | 10 | 0.000546 |
| DTT_Marius | 25 | 0.000915 | 10 | 0.000546 |
| DHH_Hector | 23 | 0.000841 | 8 | 0.000436 |
| RIX_Yolanda | 22 | 0.000805 | 7 | 0.000382 |
| DTX_unnamed | 22 | 0.000805 | 6 | 0.000327 |
| RIX_Nadine | 22 | 0.000805 | 11 | 0.000600 |
| DTM_Charon | 21 | 0.000768 | 10 | 0.000546 |
| DTC_Sherman | 21 | 0.000768 | 2 | 0.000109 |
| DTC_Eric | 18 | 0.000658 | 20 | 0.001091 |
| XXX_Sine | 17 | 0.000622 | 6 | 0.000327 |
| RLC_Danica | 17 | 0.000622 | 0 |  |
| RLX_unnamed | 17 | 0.000622 | 11 | 0.000600 |
| DTC_Alfred | 17 | 0.000622 | 7 | 0.000382 |
| DTM_unnamed | 17 | 0.000622 | 16 | 0.000873 |
| DTC_En1 | 17 | 0.000622 | 5 | 0.000273 |
| DTC_Horace | 16 | 0.000585 | 8 | 0.000436 |
| RLC_Kasia | 16 | 0.000585 | 19 | 0.001036 |
| XXX_Ecoli | 16 | 0.000585 | 4 | 0.000218 |
| RLC_HORPIA | 15 | 0.000549 | 2 | 0.000109 |
| RIX_Alexandra | 15 | 0.000549 | 27 | 0.001473 |
| DTC_DOPPIA | 15 | 0.000549 | 8 | 0.000436 |
| DTX_Nisos | 14 | 0.000512 | 11 | 0.000600 |
| RIX_Mara | 12 | 0.000439 | 6 | 0.000327 |
| DTA_unnamed | 11 | 0.000402 | 49 | 0.002673 |
| DTA_Harper | 11 | 0.000402 | 2 | 0.000109 |
| DXX_Mordred | 11 | 0.000402 | 2 | 0.000109 |
| DTT_Minimus | 10 | 0.000366 | 1 | 0.000055 |
| DXX_Hylas | 10 | 0.000366 | 0 |  |
| DTC_Seamus | 8 | 0.000293 | 0 |  |
| DTT_Xados | 7 | 0.000256 | 1 | 0.000055 |
| DTX_Erato | 6 | 0.000219 | 0 |  |
| RLG_OsTaz | 6 | 0.000219 | 2 | 0.000109 |
| DTC_Francis | 6 | 0.000219 | 2 | 0.000109 |
| DTT_Orion | 6 | 0.000219 | 0 |  |
| DTC_Isidor | 6 | 0.000219 | 0 |  |
| DTH_Pong | 6 | 0.000219 | 11 | 0.000600 |
| RIX_Sara | 5 | 0.000183 | 3 | 0.000164 |
| RLC_Lena | 5 | 0.000183 | 0 |  |
| DTX_Pegasus | 3 | 0.000110 | 1 | 0.000055 |
| RLG_Yasmin | 3 | 0.000110 | 3 | 0.000164 |
| DTC_Helios | 2 | 0.000073 | 1 | 0.000055 |
| DTX_Gorgon | 2 | 0.000073 | 2 | 0.000109 |
| DTC_Rufus | 1 | 0.000037 | 0 |  |
| DTC_Carson | 1 | 0.000037 | 0 |  |
| RIX_BALN2 | 1 | 0.000037 | 0 |  |
| DTC_Dario | 1 | 0.000037 | 0 |  |
| DTC_Irvin | 1 | 0.000037 | 0 |  |
| DTC_Eddy | 1 | 0.000037 | 0 |  |
| RIX_Wendy | 0 |  | 4 | 0.000218 |
| Novel TE families | 86459 | 3.162797 | 59832 | 3.263972 |
| Repeats (unknown) | 282130 | 10.320728 | 23419 | 1.277560 |
